# A potential acetyltransferase involved in *Leishmania major* metacaspase-dependent cell death

**DOI:** 10.1186/s13071-019-3526-4

**Published:** 2019-05-27

**Authors:** Louise Basmaciyan, Nadine Azas, Magali Casanova

**Affiliations:** 1 UMR PAM A, Valmis team, 2 rue Angélique Ducoudray, BP 37013, 21070 Dijon Cedex, France; 2 Aix Marseille Univ, IRD, AP-HM, SSA, VITROME, Marseille, France; 3 IHU-Méditerranée Infection, Marseille, France

**Keywords:** *Leishmania*, Apoptosis, Autophagy, Therapeutic target

## Abstract

**Background:**

Currently, there is no satisfactory treatment for leishmaniases, owing to the cost, mode of administration, side effects and to the increasing emergence of drug resistance. As a consequence, the proteins involved in *Leishmania* apoptosis seem a target of choice for the development of new therapeutic tools against these neglected tropical diseases. Indeed, *Leishmania* cell death, while phenotypically similar to mammalian apoptosis, is very peculiar, involving no homologue of the key mammalian apoptotic proteins such as caspases and death receptors. Furthermore, very few proteins involved in *Leishmania* apoptosis have been identified.

**Results:**

We identified a protein involved in *Leishmania* apoptosis from a library of genes overexpressed during *Leishmania* differentiation during which autophagy occurs. Indeed, the gene was overexpressed when *L. major* cell death was induced by curcumin or miltefosine. Furthermore, its overexpression increased *L. major* curcumin- and miltefosine-induced apoptosis. This gene, named *LmjF.22.0600*, whose expression is dependent on the expression of the metacaspase, another apoptotic protein, encodes a putative acetyltransferase.

**Conclusions:**

This new protein, identified as being involved in *Leishmania* apoptosis, will contribute to a better understanding of *Leishmania* death, which is needed owing to the absence of a satisfactory treatment against leishmaniases. It will also allow a better understanding of the original apoptotic pathways of eukaryotes in general, while evidence of the existence of such pathways is accumulating.

**Electronic supplementary material:**

The online version of this article (10.1186/s13071-019-3526-4) contains supplementary material, which is available to authorized users.

## Background

*Leishmania* are flagellated protozoan parasites of the family Trypanosomatidae. They are responsible, in the mammalian host, for leishmaniases that are neglected tropical diseases threatening, according to the World Health Organization, between 700,000 and 1 million people worldwide and causing between 20,000 and 30,000 deaths each year. They are transmitted by the bite of a female sand fly where they accumulate in the gut in an extracellular flagellated form called the promastigote form. In the mammalian host, promastigotes are phagocytized by macrophages where they transform into an intracellular immobile form with a much-reduced flagellum called the amastigote form. At present, there is no satisfactory treatment available for leishmaniases, owing to the cost and the parenteral mode of administration of the current drugs for people mainly living in developing countries, but also owing to the numerous side effects of many of these treatments and to the increasing emergence of drug resistance. As a consequence, new therapeutic tools are urgently required against leishmaniases. With this in mind, we decided to focus on *Leishmania* originalities, notably *Leishmania* cell death, with a view to designing new drugs targeting the parasite death in the future. Indeed, *Leishmania* cell death appears very peculiar: different stimuli can induce a form of cell death with the same phenotypic features as mammalian apoptosis (including cell shrinkage, chromatin condensation, DNA fragmentation and mitochondrial membrane depolarization) that we can call *Leishmania* apoptosis [1]. However, no key protein involved in the mammalian apoptosis, such as caspases or death receptors, could be identified in the parasite [2, 3]. Furthermore, very few proteins involved in *Leishmania* apoptosis have been identified, among which the metacaspase described as stimulating *L. major* apoptosis [4] or as a negative regulator of *L. mexicana* amastigote proliferation [5].

As part of this study, we identified a gene coding for a putative acetyltransferase, involved in *L. major* apoptosis, *LmjF.22.0600*. This gene is overexpressed under different pro-apoptotic conditions and its *in vitro* overexpression increases *L. major* curcumin- and miltefosine-induced apoptosis. Furthermore, the expression of the metacaspase is required for *LmjF.22.0600* gene expression.

## Results

### *LmjF.22.0600* and its orthologues in different *Leishmania* species

In *Leishmania*, numerous large-scale studies, involving transcriptomics and proteomics, have been conducted concerning the differentiation step from the vector promastigote form to the host cell amastigote form [6–12] during which cells undergo the cell survival process autophagy [13, 14]. Furthermore, there is a close link between apoptosis and autophagy, with accumulating evidence that, in addition to their mutual inhibition, one can activate the other. In particular, autophagy may induce apoptosis, by catabolizing parts of the cells after sequestration in autophagosomes and degradation in lysosomes, or by activating the apoptotic pathway [15]. As a consequence, we picked genes/proteins that were overexpressed during the differentiation stage and studied their involvement in *Leishmania* apoptosis. We focused on the *LinJ22_V3.0470* gene which was 3.59 times more expressed under environmental conditions of promastigote to amastigote differentiation (temperature increase up to 37 °C and pH decrease at 4.5 for 4 days) [6].

**Fig. 1:**
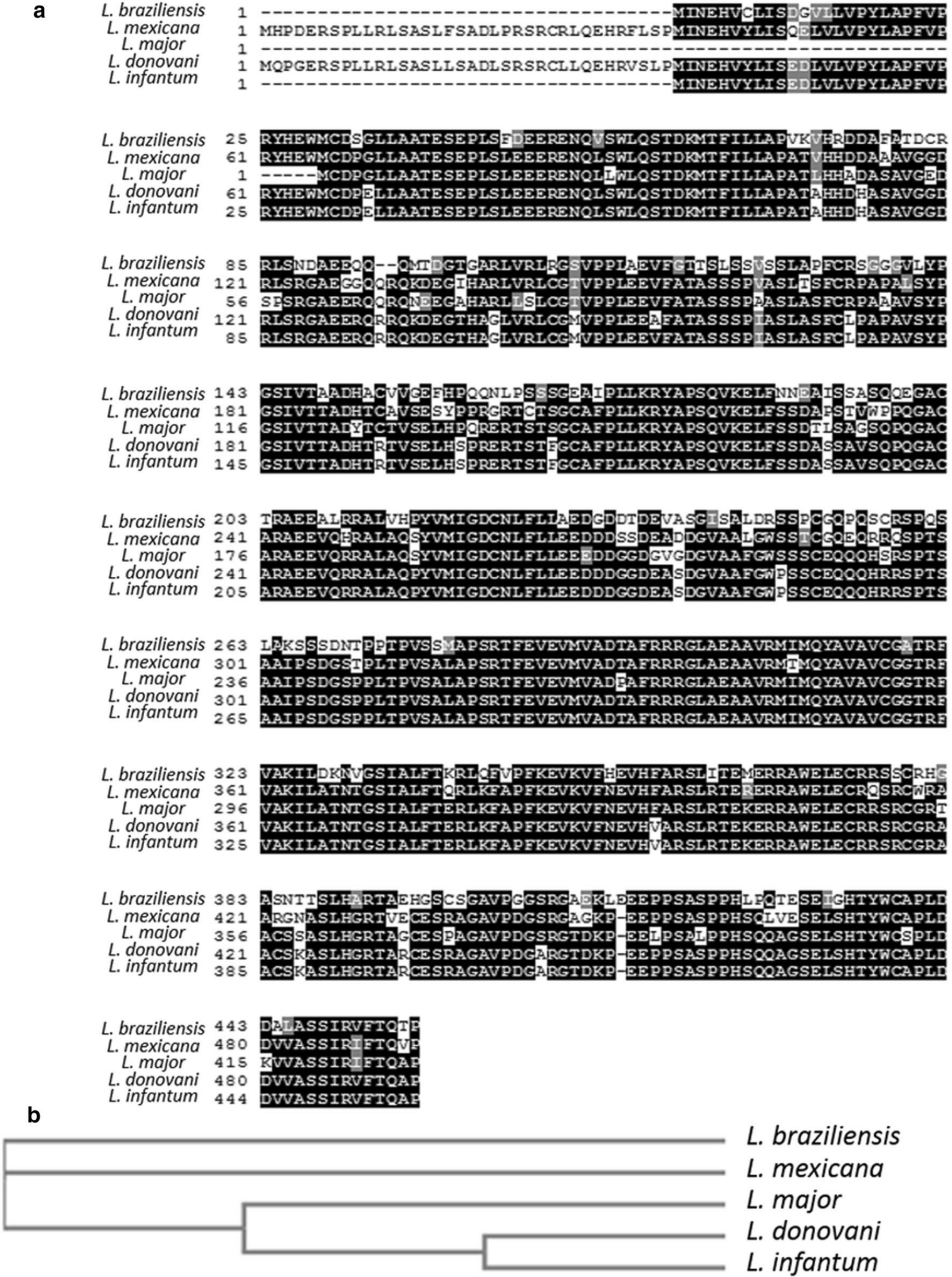
*LmjF.22.0600* and its orthologues in different *Leishmania* species. **a** Alignment of the amino acid sequences of LmjF.22.0600 orthologues in different *Leishmania* species, obtained with the Clustal Omega multiple sequence alignment software (https://www.ebi.ac.uk/Tools/msa/clustalo/) and colored with BoxShade software (https://embnet.vital-it.ch/software/BOX_form.html). **b** Cladogram of the five different LmjF.22.0600 orthologues aligned in **a**, obtained with the Clustal Omega multiple sequence alignment software

When comparing the protein sequence of different *Leishmania* species orthologues, we observed that *L. braziliensis* and *L. infantum* sequences contained an additional N-terminal domain of 24 amino-acids and *L. mexicana* and *L. donovani*, an additional N-terminal domain of 60 amino-acids (Fig. 1a). However, the protein sequence has been highly preserved among *Leishmania* species, with identity percentages ranging from 66.7% (between *L. braziliensis* and *L. major*) to 99.8% (between *L. donovani* and *L. infantum*) (Fig. 1a, b). Not surprisingly, the cladogram gathers species inducing the same form of leishmaniasis: mucocutaneous leishmaniasis for *L. braziliensis* and *L. mexicana*, visceral leishmaniasis for *L. donovani* and *L. infantum*. Orthologues have also been found in parasites from the same family: *Trypanosoma brucei* (Tb.927.7.2530) and *T. cruzi* (TcCLB.511813.10).

### The *LmjF.22.0600* gene is overexpressed during cell death induced by curcumin, miltefosine and pentamidine

**Fig. 2:**
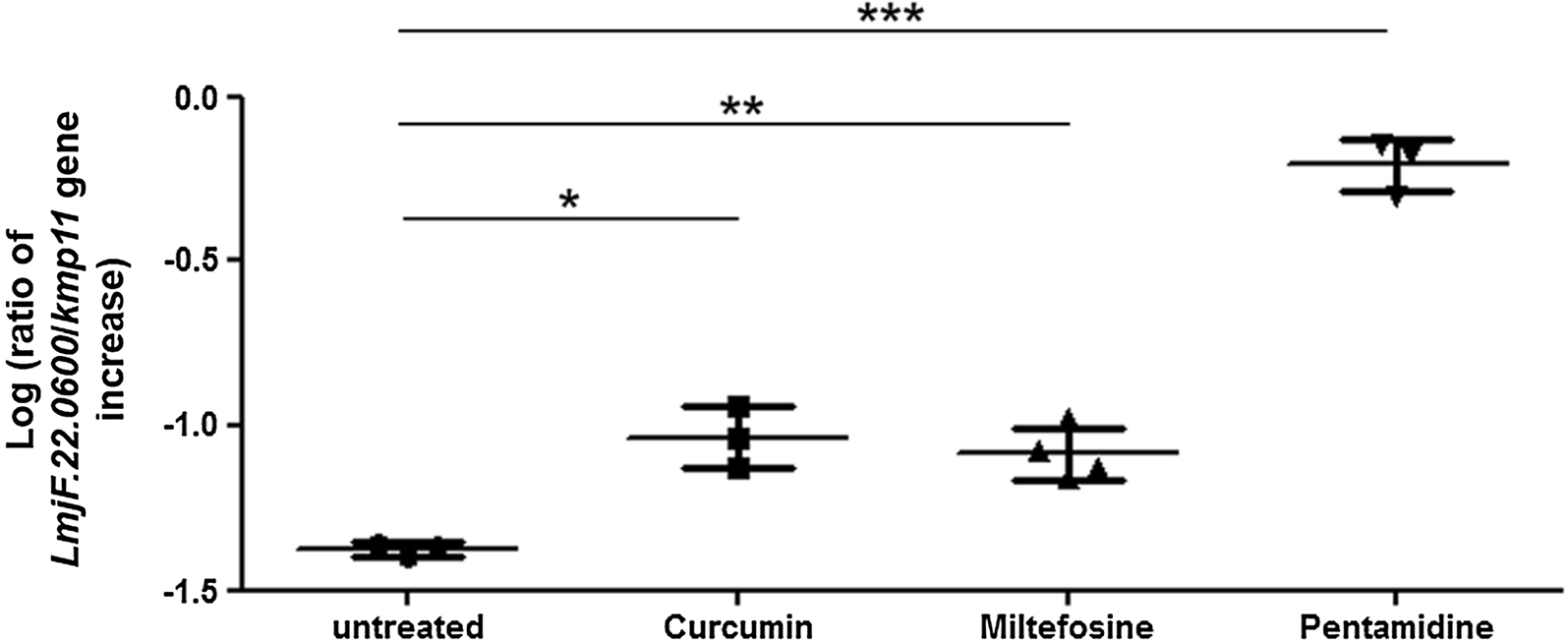
The *LmjF.22.0600* gene is overexpressed in different pro-apoptotic conditions. Ratio of expression of the *LmjF.22.0600* gene to *kmp11* control gene, represented in log, as measured by RT-qPCR, in untreated cells and cells treated for 24 h with 50 µM curcumin, 40 µM miltefosine or 100 µM pentamidine. Mean from three independent experiments for curcumin and pentamidine, and four independent experiments for miltefosine. Unpaired t-test: *P < 0.05, **P < 0.01 and ***P < 0.001

We first measured the expression of the *LmjF.22.0600* gene under pro-apoptotic conditions in log-phase promastigote *L. major* cells, in comparison to control conditions. For this, we carried out reverse-transcription quantitative PCR (RT-qPCR) on untreated cells and cells treated with curcumin, miltefosine or pentamidine. We chose these three drugs because they all induce *L. major* apoptosis [16] but through three different apoptotic pathways. Indeed, while curcumin and miltefosine respectively inhibit and activate the *L. major* metacaspase, pentamidine induces apoptosis in a metacaspase-independent manner [17]. We observed that the *LmjF.22.0600* gene was significantly overexpressed under all three apoptotic conditions compared to the housekeeping control gene *kmp11* and untreated conditions. Indeed, with curcumin and miltefosine, the expression of *LmjF.22.0600* normalized to the expression of *kmp11* was, on average, two times higher than the expression in untreated cells and it was 15 times higher in pentamidine-treated cells than in untreated cells (Fig. 2). This result indicates that *LmjF.22.0600* expression is induced under pro-apoptotic conditions in *L. major*.

### Overexpression of *LmjF.22.0600* increases curcumin- and miltefosine-induced cell death

**Fig. 3:**
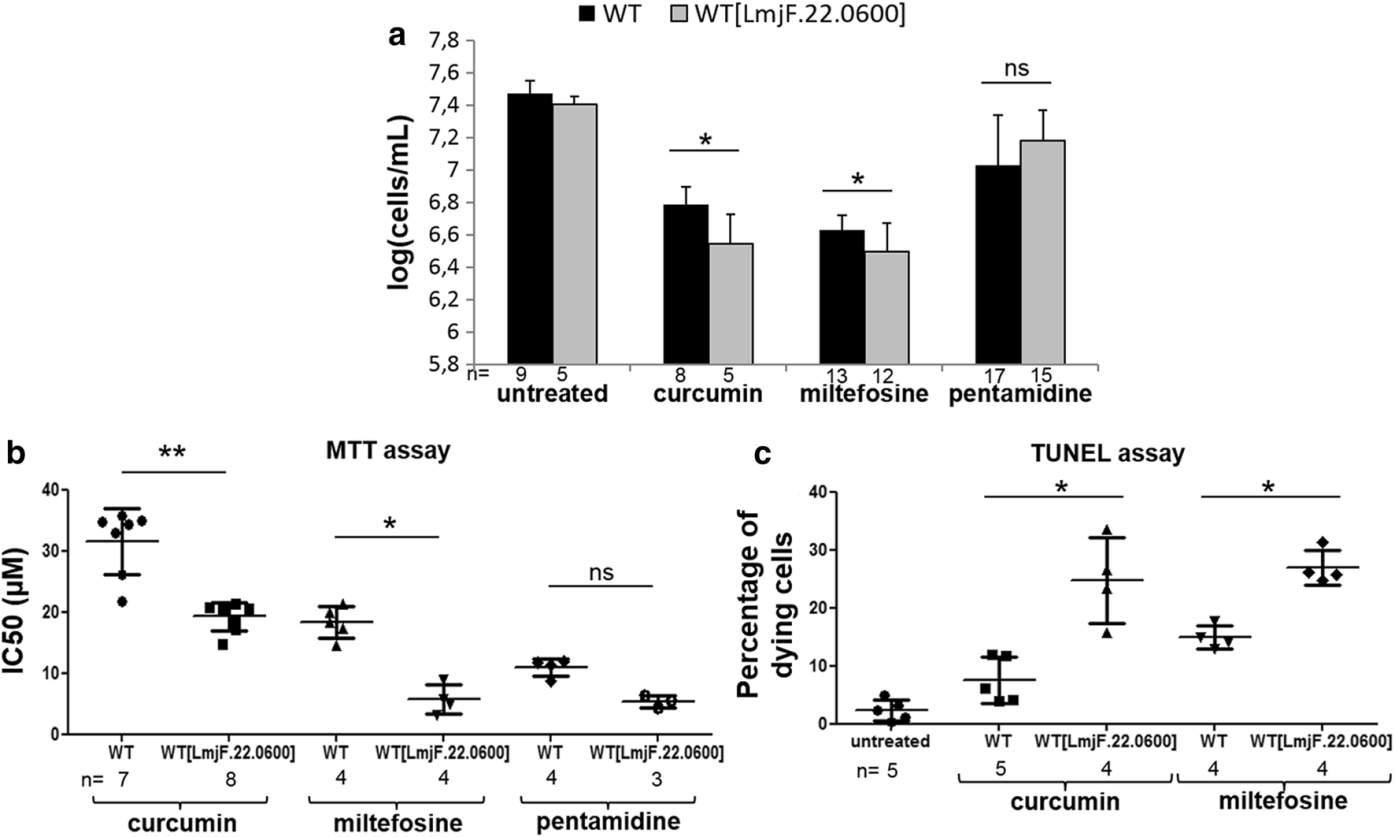
The overexpression of *LmjF.22.0600* increases curcumin- and miltefosine-induced cell death. **a** Cell concentration of WT cells and cells overexpressing *LmjF.22.0600* [WT(LmjF.22.0600)] in untreated cells and cells treated with curcumin (50 µM), miltefosine (40 µM) and pentamidine (50 µM), as measured with a hemocytometer. **b** IC50 values of curcumin, miltefosine and pentamidine in the WT strain and the strain overexpressing *LmjF.22.0600*, from MTT assays. **c** Percentage of dying cells, i.e. TUNEL-positive cells and cells without a nucleus, in the WT and *LmjF.22.0600* overexpressing strains, when left untreated or treated with 50 µM curcumin or 40 µM miltefosine for 24 h. Values are shown as the mean ± SD. The number of independent experiments that were carried out is given below the bars. Unpaired Wilcoxon-Mann-Whitney test: ns, not significant, *P < 0.05 and **P < 0.01

To confirm the role of *LmjF.22.0600* in *L. major* cell death, we overexpressed this gene by introducing it into the expression vector pTH6nGFPc and transfecting the *L. major* strain with this recombinant vector, which was maintained episomally in the cells. After the drug selection of the parasites overexpressing *LmjF.22.0600*, we verified that this overexpression did not induce any difference in cell growth compared to WT cells (see Additional file 1: Figure S1). We then studied the consequences of the overexpression under pro-apoptotic conditions. We first noted that the overexpression of *LmjF.22.0600* induced a significant decrease in cell concentration when apoptosis was induced by the addition of curcumin or miltefosine (Fig. 3a). Conversely, during pentamidine-induced cell death, overexpression of the gene had no consequence on cell concentration (Fig. 3a).

We then carried out a methyl thiazol tetrazolium (MTT) assay, which allowed us to determine the inhibitory concentration 50% (IC50) of different drugs, that is to say the concentration of drug that inhibits 50% of cell growth in comparison to the control, for the WT strain and the strain overexpressing *LmjF.22.0600*. Figure 3b shows that the IC50 of curcumin and miltefosine was significantly lower in the strain overexpressing *LmjF.22.0600* than in the WT strain. Concerning pentamidine, no significant difference in the IC50 was observed between the WT and the recombinant strain (Fig. 3b). These results, from cell counting and IC50, show the involvement of LmjF.22.0600 in *L. major* curcumin- and miltefosine-induced cell death.

In order to clarify the type of cell death induced by curcumin and miltefosine when *LmjF.22.0600* was overexpressed, we carried out a TUNEL assay to detect DNA fragmentation, a clear characteristics of apoptotic cells, even in the *Leishmania* parasite [16]. In this assay, we measured the percentage of dying cells, i.e. the percentage of cells with a TUNEL-positive nucleus and also of cells that no longer had a nucleus. The significant increase in the percentage of dying cells with curcumin and miltefosine, when *LmjF.22.0600* was overexpressed, in comparison to WT cells, indicates that LmjF.22.0600 increased *L. major* curcumin- and miltefosine-induced apoptosis (Fig. 3c).

### The expression of *LmjF.22.0600* is inhibited in the metacaspase-deleted strain

**Fig. 4:**
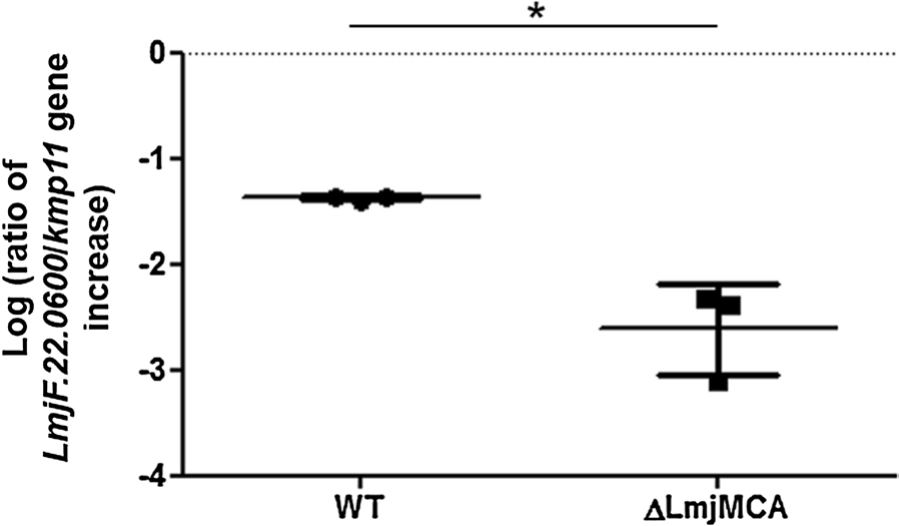
The expression of *LmjF.22.0600* is inhibited in the metacaspase-deleted strain. RT-qPCR quantification of *LmjF.22.0600* expression normalized to the expression of the control gene *kmp11*, in the WT and LmjMCA-deleted strains (logarithmic scale). Values are shown as the mean ± SD from three independent experiments. Unpaired t-test: *P < 0.05

We measured by RT-qPCR the expression of *LmjF.22.0600* in the WT strain and in the strain deleted from the *L. major* metacaspase LmjMCA. We observed that *LmjF.22.0600* expression, normalized to the expression of the *kmp11* control gene, was significantly inhibited in the LmjMCA-deleted strain (Fig. 4). On average, *LmjF.22.0600* expression, normalized to *kmp11* expression, was 13 times smaller in the LmjMCA-deleted strain than in the WT strain. Yet, the *LmjF.22.0600* gene was present in the genome of both strains, as confirmed by PCR analysis (see Additional file 2: Figure S2). As a consequence, the expression of LmjMCA is required for expression of the *Lmj.22.0600* gene.

## Discussion

In this study, we identified a protein called LmjF.22.0600 involved in *L. major* apoptosis, the corresponding gene being overexpressed after the addition of different pro-apoptotic drugs and its overexpression increasing curcumin- and miltefosine-induced cell death. We can note that the inhibition of the expression of the gene by CRISPR/Cas9 gave unexpected results. More precisely, while it induced a higher cell concentration compared to the WT cells after the addition of curcumin, it induced no significant difference in cell concentration after the addition of miltefosine (see Additional file 3: Figure S3a). Furthermore, co-staining with calcein and propidium iodide (PI), *Leishmania* apoptotic markers [18], did not allow us to detect any apoptosis inhibition or activation when the gene was deleted (see Additional file 3: Figure S3b, c). However, in *Leishmania*, cell death is essential for avoiding hyperparasitism by regulating the parasite cell density in the sand fly vector and in the mammalian host [19], for eliminating unfit cells and therefore allowing the fittest cells to survive [20], and for modulating host immunity, for instance by inducing the release of anti-inflammatory cytokines and the downregulation of pro-inflammatory cytokines, which would favor parasite survival [19]. Owing to the importance of apoptosis in the parasite, we can hypothesize that the absence of effect of LmjF.22.0600 deletion would be due to the involvement of other protein(s) that would take over in the apoptotic pathway. *LmjF.22.0600* encodes a potential acetyltransferase that would induce post-translational modifications participating in *L. major* apoptosis. The protein LmjF.22.0600 is involved in the same apoptotic pathway as the metacaspase LmjMCA. Indeed, the expression of the metacaspase is required for *LmjF.22.0600* expression. Furthermore, LmjF.22.0600 overexpression had no consequence on pentamidine-induced *L. major* cell death, whereas pentamidine induces apoptosis through a metacaspase-independent pathway [17]. Our hypothesis is that the pro-apoptotic drug miltefosine induces the catalytic activation of LmjMCA, inducing *LmjF.22.0600* gene expression activation, LmjF.22.0600 protein overexpression, and *Leishmania* apoptosis *via* proteins that are currently unknown. This constitutes the sixth protein identified as being involved in *Leishmania* apoptosis, with the metacaspase [4], the endonuclease G [21, 22], the cysteine proteinase C [23], the pro-apoptotic molecule with a BH3 domain Li-BH3AQP [24] and the hydrolase LmjHYD36 [25]. These six proteins involved in *Leishmania* apoptotic pathways are at variance with the idea of incidental death [3]. On the contrary, it is in concordance with the idea of programmed (physiological) or regulated (non physiological) cell death in this unicellular parasite.

*LmjF.22.0600* was identified from a library of genes overexpressed during the *Leishmania* promastigote to amastigote differentiation process during which autophagy occurs. As a consequence, while many experiments have been carried out on this step, it is an important source of genes/proteins potentially involved in *Leishmania* apoptosis. Through this approach, many genes other than *LmjF.22.0600*, coding proteins involved in *Leishmania* apoptosis, could therefore be identified, such as the recently published gene *LmjF.36.6540* coding a potential hydrolase [25]. Here, the experiments have been carried out *in vitro* and on the promastigote *L. major* forms, using drugs as apoptotic inducers. Therefore, we can wonder whether the identified protein is also involved *in vivo* in *Leishmania* apoptosis within the sand fly gut or within the mammalian host.

## Conclusions

The identification of additional genes involved in *Leishmania* apoptosis, even *in vitro*, contributes to a better understanding of original apoptotic pathways (notably caspase-independent) of eukaryotes in general, while evidence of the existence of such pathways is accumulating [26]. It also contributes to a better understanding of *Leishmania* biology, an essential element for the future design of new drugs to treat the neglected tropical diseases, leishmaniases, for which there are no satisfactory treatments.

## Methods

### Parasites

For *L. major* ‘Friedlin’ promastigotes (MHOM/IL/81/Friedlin) culture, 10^6^ cells/ml were routinely seeded in Schneider’s *Drosophila* medium (Thermo Fisher Scientific, Waltham, MA, USA) supplemented with 100 U/ml penicillin, 100 µg/ml streptomycin, 2 mM glutamine and 20% fetal calf serum (Thermo Fisher Scientific) at 26 °C for 48 h before subculturing when reaching 10^7^ cells/ml.

### Induction of cell death

Logarithmic *L. major* cells were harvested by centrifugation and incubated at 10^6^ cells/ml for the MTT assays or at 10^7^ cells/ml for all other experiments, with 30 or 50 µM curcumin (Sigma-Aldrich, Saint-Louis, MO, USA), 40 µM miltefosine (Santa Cruz Biotechnology, Dallas, TX, USA) or 50 or 100 µM pentamidine (Sigma-Aldrich), drug concentrations previously shown as inducing *L. major* apoptosis [16].

### Reverse-transcription quantitative PCR

For RNA extraction, the RNeasy Plus mini kit (Qiagen, Courtaboeuf, France) was used according to the manufacturer’s procedure, the final RNA concentration being evaluated with a Nanodrop 2000c spectrophotometer (Thermo Fisher Scientific). One-step reverse transcription was performed using the high capacity cDNA reverse transcription kit (Applied Biosystems, Foster City, CA, USA) according to the manufacturer’s instructions. For the quantitative PCR, 5 µl of cDNA was added to 20 µl of PCR mix containing Sybr Green I (Roche, Meylan, France) and placed in a Light Cycler 480 (Roche) with the following cycling conditions: Taq polymerase activation at 95 °C for 10 min then 45 cycles of amplification of 95 °C for 15 s and 60 °C for 60 s. The *kmp11* (kinetoplastid membrane protein 11) gene was used as a control. Gene expression was calculated using the Pfaffl method in which the efficiency of each pair of primers is used, after its determination using the serial dilution method on the basis of a linear regression slope [27]. The oligonucleotides used in this study are listed in Additional file 4: Table S1.

### Construction of the *LmjF.22.0600*-overexpressing strain

For the construction of the *LmjF.22.0600*-overexpressing strain, *LmjF.22.0600* was PCR-amplified from *L. major* genomic DNA. The PCR product was cloned into pGEM-T-Easy (Promega, Madison, WI, USA) and then inserted into the expression vector pTH6nGFPc (kind gift from Patrick Bastien, Montpellier University) after digestion with MfeI and HpaI restriction enzymes, vector that places the GFP sequence at the 3’-end of the *LmjF.22.0600* sequence. The primers used are listed in Additional file 4: Table S1.

Deletion of the *LmjF.22.0600* gene was performed as described in the article of Beneke et al. [28]. The primers were designed thanks to the online tool developed by the authors: http://leishgedit.net/ (see Additional file 4: Table S1). For the PCR-amplification of the donor DNA, 20 ng of plasmids LC100 and LC101 (kind gift from Eva Gluenz, University of Oxford), 0.4 mM dNTP, 0.5 µM each of gene-specific forward and reverse primers and 0.5 µl of Phusion High-Fidelity DNA Polymerase (New England Biolabs, Ipswich, MA, USA) were mixed in a final volume of 50 µl. To amplify the guide RNAs, 0.6 mM dNTP, 2 µM each of gene-specific forward and reverse primers and 0.5 µl of Phusion High-Fidelity DNA Polymerase were mixed in a final volume of 50 µl. The PCR conditions were as follows: 30 s at 98 °C, then 40 cycles of 10 s at 98 °C, 30 s at 62 °C and 1 min at 72 °C, with a final elongation step of 10 min at 72 °C. After assessing the presence of the expected PCR products by migration on an agarose gel, the PCR products (each PCR was done in duplicate) were pooled and purified with the Wizard SV Gel and PCR Clean-Up System (Promega). The purified PCR products were heat sterilized at 95 °C for 5 min prior to transfection.

### Transfection procedure

For the *LmjF.22.0600* overexpression, 3 × 10^7^ *L. major* cells in logarithmic phase were transfected with 10 µg of the recombinant vector pTH6nGFPc containing the *LmjF.22.0600* sequence, in 100 µl of Human T Cell Nucleofector solution (Lonza, Basel, Switzerland).

For the *LmjF.22.0600* deletion, 10^7^ *L. major* cells expressing Cas9 and T7 (kind gift from Eva Gluenz, University of Oxford) in logarithmic phase were transfected with 30 µg of purified PCR. The transfection buffer was composed of 90 mM sodium phosphate, 5 mM potassium chloride, 0.15 mM calcium chloride, 50 mM HEPES, pH 7.3. The same transfection without purified PCR was used as control.

The transfections were performed in 2 mm gap cuvettes (Lonza) with program X-001 of the Amaxa Nucleofector II (Lonza). Transfected cells were immediately transferred into 5 ml pre-warmed medium and left to recover overnight at 26 °C before adding 30 µg/ml hygromycin B (Thermo Fisher Scientific) for the overexpression and two drugs for the deletion: geneticin (Sigma-Aldrich) at 20 µg/ml and puromycin (Sigma-Aldrich) at 30 µg/ml.

### MTT assay

WT *L. major* cells or cells overexpressing *LmjF.22.0600* were incubated in triplicate at 10^6^ cells/ml with various concentrations of curcumin, miltefosine or pentamidine for 72 h. As controls, cells were also incubated with ethanol (curcumin solvent) and H_2_O (miltefosine and pentamidine solvent). MTT at 5 mg/ml (20 µl) was then added. Cells were further incubated for 4 h at 26 °C and 100 µl of a blocking solution was added (10% m/v sodium dodecyl sulfate and 50% v/v isopropanol). Optical density, measured with a spectrophotometer (ELX808 Ultra Microplate Reader; Bio-Tek Instruments, Colmar, France) at 570 nm, allowed us to determine the drug IC50, the drug concentration inhibiting 50% of cell growth compared to the control.

### TUNEL assay

The *In Situ* Cell Death Detection Kit, Fluorescein (Roche), was used for detecting DNA fragmentation characteristics of apoptotic cells by fluorescence microscopy. To do so, cells were fixed with formaldehyde 4%, laid on an immunoslide and permeabilized with a 0.1% triton and 0.1% sodium citrate solution. The reaction solution from the kit, with the enzyme diluted 1/10, was then added, before observation with a BX51 fluorescence microscope (Olympus, Rungis, France). Bright field and fluorescence images were acquired using the fluorescence imaging system Cell^A^ (Olympus).

### Calcein and PI labeling

Cells were washed once in PBS and resuspended in 1 ml of PBS containing 2 µl of calcein (LIVE/DEAD® Viability/Cytotoxicity Kit for mammalian cells; Molecular Probes, Eugene, OR, USA) diluted 1/80 in DMSO and 5 µl of PI at 0.5 mg/ml. The mixed sample was then incubated for 15–20 min at room temperature and protected from light. The cells were analyzed by flow cytometry using an excitation wavelength of 488 nm and measuring green fluorescence emission for calcein (530/30 nm bandpass) and red fluorescence emission for PI (610/20 nm bandpass) on a BD LSRFortessa™ cell analyzer (BD, Le Pont de Claix, France). Data were exported and analyzed with Flowjo software for evaluation of the percentage of calcein- and PI-positive cells.

### Statistical analysis

For statistical analysis, unpaired Wilcoxon-Mann-Whitney tests or t-tests were performed with BioStaTGV (https://biostatgv.sentiweb.fr/?module=tests). Results, obtained from a minimum of three independent experiments, were considered statistically significant when *P* < 0.05; otherwise, “ns” was written, for not significant. For significant differences: *P < 0.05, **P < 0.01 and ***P < 0.001.

## Additional files

**Additional file 1: Figure S1.** The overexpression of LmjF.22.0600 induces no growth defect. Growth curves of the WT and *LmjF.22.0600*-overexpressing [WT(LmjF.22.0600)] cells: *n* ≥ 3.

**Additional file 2: Figure S2.** The *LmjF.22.0600* gene is present in WT and LmjMCA deleted cells. Agarose gel electrophoresis after a PCR with *LmjF.22.0600*-specific primers, showing the presence of the *LmjF.22.0600* gene in the WT cell line as well as in the LmjMCA-deleted cells (ΔLmjMCA).

**Additional file 3: Figure S3.** The inhibition of *LmjF.22.0600* expression by CRISPR/Cas9 induces almost no change concerning *L. major* apoptosis. **a** Cell concentration of WT and *LmjF.22.0600*-deleted cells after curcumin (30 µM), miltefosine (40 µM) and pentamidine (100 µM) induced cell death, as measured with a hemocytometer. Values are shown as the mean ± SD. The number of independent experiments is written in the figure. **b** Percentage of calcein-positive WT and *LmjF.22.0600*-deleted cells after the induction of *L. major* apoptosis with 30 µM curcumin, 40 µM miltefosine or 100 µM pentamidine. No significant difference was observed between the WT and the deleted strains, according to a Mann-Whitney test. **c** Percentage of PI-positive WT and *LmjF.22.0600*-deleted cells after the induction of *L. major* apoptosis with 30 µM curcumin, 40 µM miltefosine or 100 µM pentamidine. No significant difference was observed between the WT and the deleted strains, according to a Mann-Whitney test.

**Additional file 4: Table S1.** Oligonucleotides used for RT-qPCR, *LmjF.22.0600* overexpression and *LmjF.22.0600* deletion.

## Abbreviations

GFP: green fluorescent protein
IC50: inhibitory concentration 50%
MCA: metacaspase
MTT: methyl thiazol tetrazolium
PI: propidium iodide
RT-qPCR: reverse-transcription quantitative PCR

## Availability of data and materials

All data generated or analyzed during this study are provided within the article and its additional files or are available from the corresponding author upon request.
