# Supplementary figures and images for: A potential acetyltransferase involved in Leishmania major metacaspase-dependent cell death
Source: Parasit Vectors. 2019 May 27;12:266. doi: 10.1186/s13071-019-3526-4 (PMC6537415; doi:10.1186/s13071-019-3526-4)

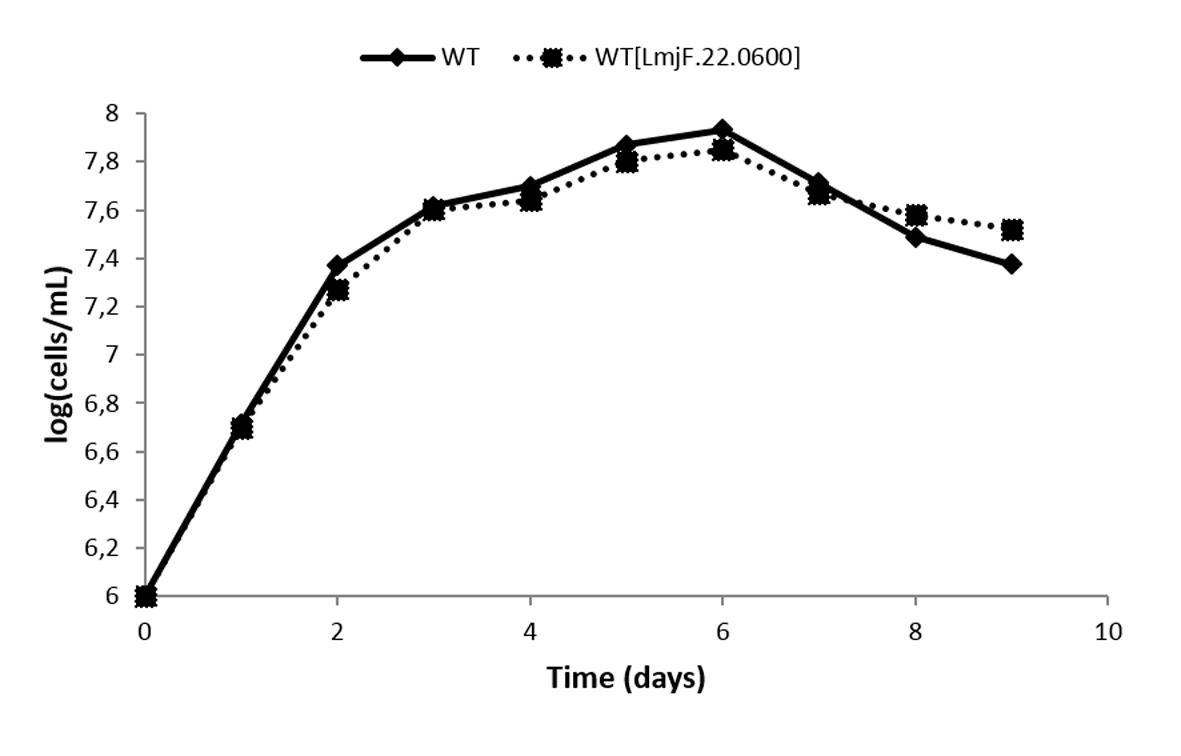

Supplement: Supplementary file 1 — Additional file 1: Figure S1. The overexpression of LmjF.22.0600 induces no growth defect. Growth curves of the WT and LmjF.22.0600-overexpressing [WT(LmjF.22.0600)] cells: n ≥ 3. [file 13071_2019_3526_MOESM1_ESM.tif]

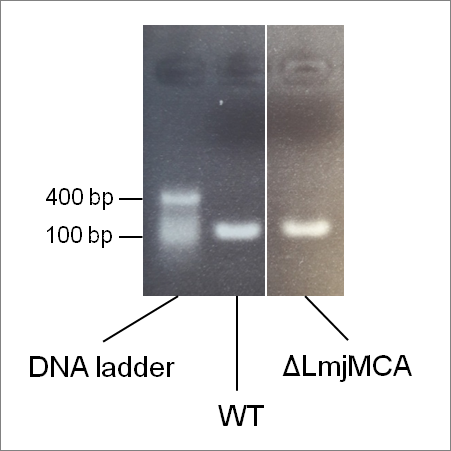

Supplement: Supplementary file 2 — Additional file 2: Figure S2. The LmjF.22.0600 gene is present in WT and LmjMCA deleted cells. Agarose gel electrophoresis after a PCR with LmjF.22.0600-specific primers, showing the presence of the LmjF.22.0600 gene in the WT cell line as well as in the LmjMCA-deleted cells (ΔLmjMCA). [file 13071_2019_3526_MOESM2_ESM.tif]

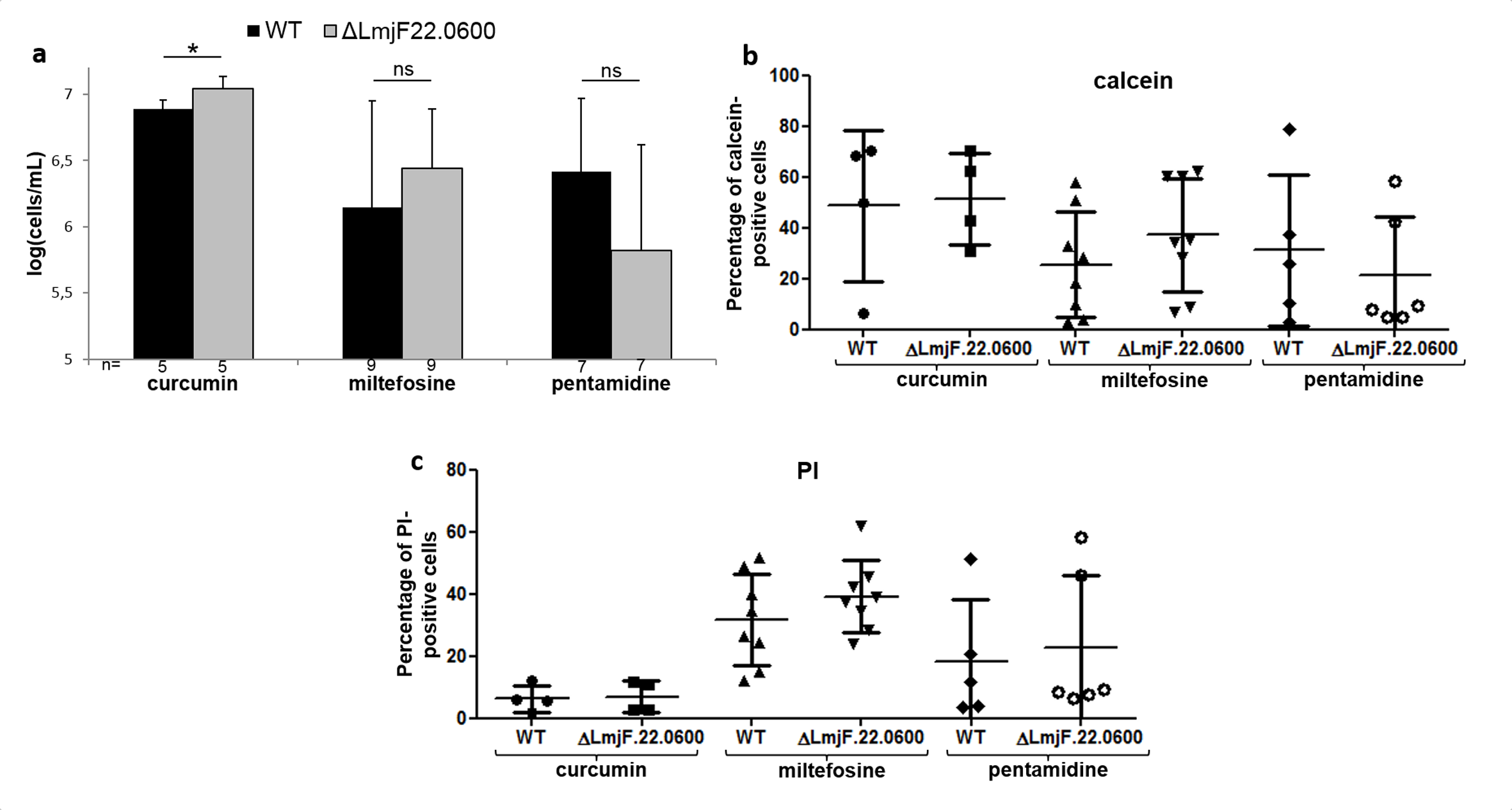

Supplement: Supplementary file 3 — Additional file 3: Figure S3. The inhibition of LmjF.22.0600 expression by CRISPR/Cas9 induces almost no change concerning L. major apoptosis. a Cell concentration of WT and LmjF.22.0600-deleted cells after curcumin (30 µM), miltefosine (40 µM) and pentamidine (100 µM) induced cell death, as measured with a hemocytometer. Values are shown as the mean ± SD. The number of independent experiments is written in the figure. b Percentage of calcein-positive WT and LmjF.22.0600-deleted cells after the induction of L. major apoptosis with 30 µM curcumin, 40 µM miltefosine or 100 µM pentamidine. No significant difference was observed between the WT and the deleted strains, according to a Mann-Whitney test. c Percentage of PI-positive WT and LmjF.22.0600-deleted cells after the induction of L. major apoptosis with 30 µM curcumin, 40 µM miltefosine or 100 µM pentamidine. No significant difference was observed between the WT and the deleted strains, according to a Mann-Whitney test. [file 13071_2019_3526_MOESM3_ESM.tif]
