# Supplementary material for: A potential acetyltransferase involved in Leishmania major metacaspase-dependent cell death
Source: Parasit Vectors. 2019 May 27;12:266. doi: 10.1186/s13071-019-3526-4 (PMC6537415; doi:10.1186/s13071-019-3526-4)
Supplement: Supplementary file 4 — Additional file 4: Table S1. Oligonucleotides used for RT-qPCR, LmjF.22.0600 overexpression and LmjF.22.0600 deletion. [file 13071_2019_3526_MOESM4_ESM.doc]

**Additional file 4. Table S1.** Oligonucleotides used for RT-qPCR, *LmjF.22.0600* overexpression and *LmjF.22.0600* deletion.

| **Construction** | **Oligonucleotide name** | **Oligonucleotide sequence** |
| --- | --- | --- |
| RT-qPCR | dRTLm22.0600 | TCGAAGAGGTGTTTGCGACA |
|  | rRTLm22.0600 | CGTGTAATCTGCGGTGGTGA |
| *LmjF.22.0600* overexpression | dCLm22.0600pTH6 | GGGCAATTGATGCAGCCAGGCGAGCG |
|  | rCLm22.0600pTH6 | GGGGTTAACTGGCGCTTGTGTGAAGATC |
| *LmjF.22.0600* deletion | UpFLmj220600 | CTGTCTCAACTGGCGACGTGCTTCACGCCTgtataatgcagacctgctgc |
|  | DownRLmj220600 | CCTTATCTGCCCCCCTCCCTGCCGCGCTGCccaatttgagagacctgtgc |
|  | 5'sgRNALmj220600 | gaaattaatacgactcactataggACGGCGAAACCACAACCGACgttttagagctagaaatagc |
|  | 3'sgRNALmj220600 | gaaattaatacgactcactataggCGCTGCAGTTGGCTACGTCGgttttagagctagaaatagc |
|  | sgRNA reverse | AAAAGCACCGACTCGGTGCCACTTTTTCAAGTTGATAACGGACTAGCCTTATTTTAACTTGCTATTTCTAGCTCTAAAAC |
